# Research progress on the mechanism of probiotics regulating cow milk allergy in early childhood and its application in hypoallergenic infant formula

**DOI:** 10.3389/fnut.2024.1254979

**Published:** 2024-02-14

**Authors:** Mao Lin, Cong Yanjun

**Affiliations:** Beijing Advanced Innovation Center for Food Nutrition and Human Health, Beijing Engineering and Technology Research Center of Food Additives, College of Food and Health, Beijing Technology and Business University, Beijing, China

**Keywords:** cow's milk allergy, probiotics, regulation, hypoallergenic infant formula, mechanism

## Abstract

Some infants and young children suffer from cow's milk allergy (CMA), and have always mainly used hypoallergenic infant formula as a substitute for breast milk, but some of these formulas can still cause allergic reactions. In recent years, it has been found that probiotic nutritional interventions can regulate CMA in children. Scientific and reasonable application of probiotics to hypoallergenic infant formula is the key research direction in the future. This paper discusses the mechanism and clinical symptoms of CMA in children. This review critically ex- amines the issue of how probiotics use intestinal flora as the main vector to combine with the immune system to exert physiological functions to intervene CMA in children, with a particular focus on four mechanisms: promoting the early establishment of intestinal microecological balance, regulating the body's immunity and alleviating allergic response, enhancing the intestinal mucosal barrier function, and destroying allergen epitopes. Additionally, it overviews the development process of hypoallergenic infant formula and the research progress of probiotics in hypoallergenic infant formula. The article also offers suggestions and outlines potential future research directions and ideas in this field.

## 1 Introduction

Food allergy refers to the abnormal immune reaction to food proteins, which leads to the disorder of physiological function or tissue damage of the body, thus causing a series of clinical symptoms (1). According to the statistics of the World Health Organization (WHO), at present, food allergic reaction has risen to the sixth place in the global diseases, and the number of people suffering from such diseases has increased exponentially (2), affecting more than 20% of the world's population, especially children (3), and becoming the most important non-infectious disease affecting children's health. “Big-8 allergenic foods” had been identified, including gluten containing grains, crustaceans, fish, eggs, peanuts, soybeans, milk, nuts and products of the above 8 categories of substances (4). A series of investigation results show that the early life allergic reaction is mainly milk, egg allergy (5–11).

CMA is an allergic immune response to cow milk protein (CMP) that usually develops in the first few months after birth (12, 13). Cow's milk is an important source of nutrients when breastfeeding is insufficient (14). For children with CMA, different types of hydrolyzed formulas (HF) are recommended, extensively hydrolyzed formula (eHF) as the first choice for CMA treatment, and amino acid formulas (AAF) for more severe cases or those with reaction to eHF (15–18). In recent years, the infant formula adding probiotics were developed, and whether probiotics can reduce the risk of CMA, the present manuscript summarizes and discusses the mechanism and application of probiotics in early life to regulate CMA in children.

## 2 Mechanism and clinical symptoms of CMA in children

Cow milk contains large allergenic proteins. Most important of them are casein, β-lactoglobulin (BLG), and α-lactalbumin (19). CMA is one of the most common food allergies and ranks third among all food allergies leading to anaphylaxis (8–15% of cases) especially in childhood, affecting about 3–8% of children in different countries (20, 21). Infants are prone to CMA, which is mainly caused by the immature development of intestinal barrier and the incomplete development of immune system (22, 23). The intestinal mucosal cells of infants are sparsely arranged, the intestinal osmotic pressure is increased, and allergens are easy to enter the blood through mucosal cells to cause allergy.

**Figure 1:**
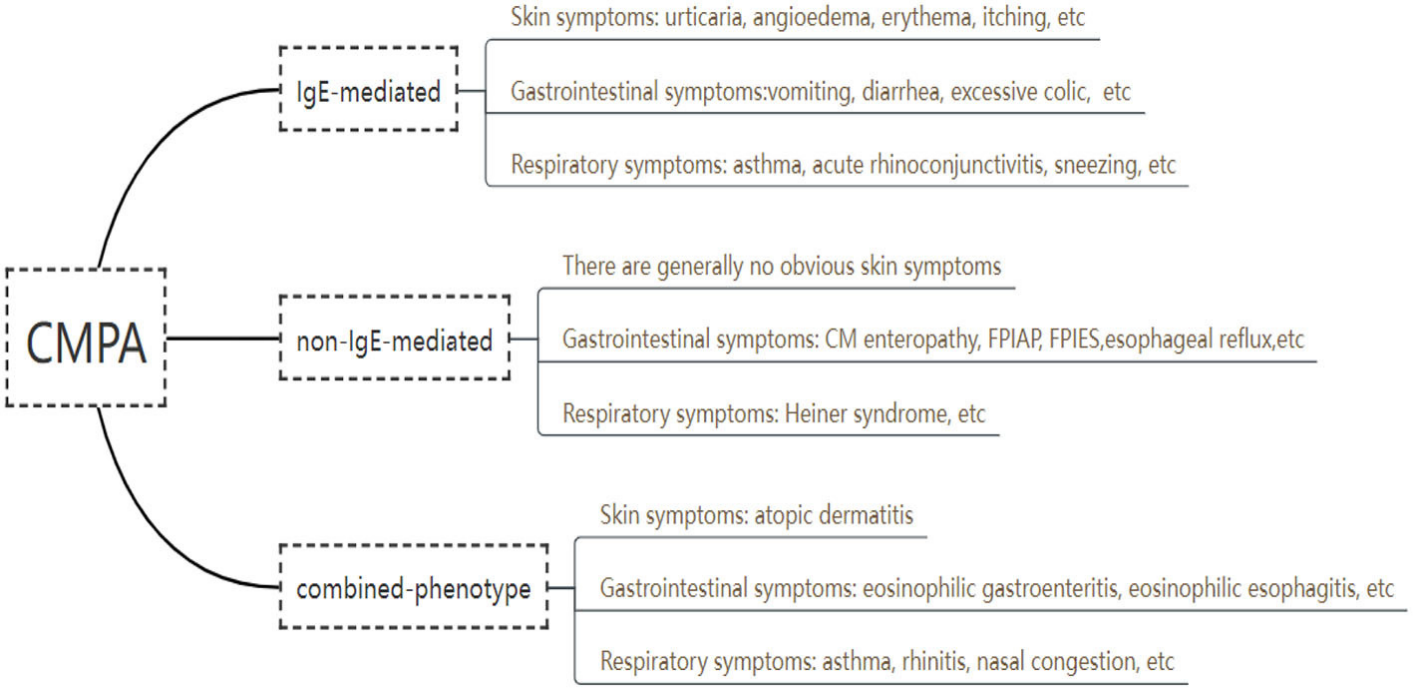
Clinical symptoms of cow's milk allergy in children.

Based on immunological mechanisms, CMA can be divided into three types, including immunoglobulin E (IgE) -mediated, non IgE -mediated, and combined (Figure 1). In a Brazilian referral center of Allergy and Clinical Immunology, clinical history, laboratorial findings and test results were collected from 115 pediatric patients with CMA in 2017 through electronic medical record, the results showed that 57% of the reactions were IgE-mediated, 20% were non-IgE-mediated and 23%, mixed reactions (24). An IgE-mediated CMA is a type I hypersensitivity reaction or immediate CMA, and the clinical manifestations occur within minutes to 2 h after milk ingestions, which involves mast cell degranulation (25). Tang et al. shown that among the 234 participants who were measured by an allergen array, 9 were boiled milk sIgE-positive, 50 were yogurt sIgE-positive, 17 were buttermilk sIgE-positive, and 158 were only raw milk sIgE-positive (26). Non IgE-mediated CMA often present symptoms 2 h to even several days induced by exposure to cow milk, involving respiratory tract, gastrointestinal tract and other parts (27, 28), including type II or type III hypersensitivity reactions mediated by IgG or IgM and tissue damage caused by complement, basophils and neutrophils, and type IV hypersensitivity reactions mediated by T lymphocytes (29). The allergic mechanism of non-IgE-mediated immune response is currently under debate and still needs further research. Combined CMA may be related to the cross-inhibitory response of Th1 and Th2 in the immune system of newborn infants (30). This cross-inhibitory response may have humoral and/or cell-mediated mechanisms and may present with symptoms such as atopic dermatitis, allergic eosinophilic esophagitis, and eosinophilic gastritis.

In recent years, more and more studies have shown that the imbalance of Treg and pro-inflammatory Th17 cells (Treg/Th17) is also one of the key factors causing allergic diseases. When milk protein allergic reaction occurs, Th17 is dominant, and the number of Treg decreases (31).

## 3 The development of hypoallergenic infant formula

Hypoallergenic foods are those that are well tolerated in at least 90% (95% of confidence interval) individuals with allergies in double-blind, placebo-controlled trials (32, 33). Hypoallergenic infant formula is a kind of infant formula for special medical use, and it can be divided into partially hydrolyzed formulas (pHF), eHF, and AAF (see Table 1) (34). Hydrolysis may destroy the epitopes of CMA by hydrolyzing part or all of the milk proteins into small molecular peptides and amino acids, which reduces the antigenicity of CMP. pHF is mainly used for dietary management of infants with functional gastrointestinal disorders and can also be used for initial intervention feeding of non-breastfed infants at high risk of milk protein allergy (whose parents or siblings have a history of allergy). eHF and AAF are mainly used in the dietary management of infants with CMA (35–39).

**Table 1:** Classification and characteristics of infant formula for special medical purposes (34).

**Type**	**Molecular size**	**Main characteristics of the formula**	**Effectiveness of immunization**
pHF	< 5,000 Da	1. Some epitopes of the allergen remain. 2. Peptides of sufficient size were retained to stimulate the induction of oral tolerance based on deeply hydrolyzed milk protein formulations	1. It is beneficial to the normal development of intestinal tract 2. Ingestion of cow's milk protein over a period of time can stimulate infant tolerance to cow's milk protein
eHF	< 1,500 Da	1. The antigenicity is reduced, and the molecular mass is small 2. Trace amounts of allergens were present	1. It reduces the allergic reaction and stimulates the development of immune tolerance 2. Long-term consumption affects the development of intestinal function in infants, and gastrointestinal reactions still occur
AAF	< 500 Da	1. Based on oligopeptide and purified amino acid, the effect of milk allergens can be avoided 2. It contains normal carbohydrates and fats, providing nutrients for infant growth and development	1. It has a significant improvement effect on the condition 2. It is not clear whether immune tolerance to cow's milk protein can be established

In some pHF, B cell epitopes of cow milk allergens are still present, which can cause CMA (40). It has been shown that there is a significant difference in BLG residues between pHF and eHF. The BLG level of pHF is 40,000 times higher than that of eHF (41). pHF seems to be a better alternative to infant formula based on CMP (42). However, pHF outperforms most eHF in terms of cost and taste preference (43). Although there is no clear definition of eHF and pHF, these two infant formulas have been developed and commercially available. Hydrolyzed infant formula varies due to protein source, degree of hydrolysis, protease species, auxiliary processing techniques (such as thermal processing) and peptide profile, and allergen residues may still be present (42). The antigenicity of allergen residues in infant formula depends on the degree of hydrolysis and filtration techniques applied during the preparation; therefore, it is recommended that the safety of the hydrolyzed infant formula be first confirmed before it is introduced into the diet of CMA infants (41). The main criterion for labeling infant formula as hypoallergenic is that 90% of children or infants with CMA confirmed by double-blind, placebo-controlled trials do not exhibit allergic reactions (44).

Based on pHF and eHF research, AAF was subsequently developed. Studies have shown that the use of AAF can greatly reduce sensitization reactions (45). AAF supplementation is recommended for infants with pHF or eHF allergy, growth retardation, or multiple food allergies (45). The main problem with AAF not being widely applied is the high cost and rather unpleasant taste (46). In some studies, eHFs, and AAFs were noted to be hypoallergenic, while pHFs was not included because the allergic reaction caused by it was unpredictable (47).

Up to now, the impact of intestinal flora diversity and/or dysfunction (dysbiosis) on food allergy has attracted more people's attention, and the addition of probiotics to infant formula to develop new products has become a research hotspot. At present, there are mainly the following probiotics that are permitted to be used in infant food in China, such as *Lactobacillus acidophilus* NCFM, *Bifidobacterium animalis subsp. Lactis* Bb-12, *Bifidobacterium animalis subsp. Lactis* HN019, *Bifidobacterium animalis subsp. Lactis* Bi-07, *Lacticaseibacillus rhamnosus* GG (LGG), *Lacticaseibacillus rhamnosus* HN001*, Lacticaseibacillus rhamnosus* MP108*, Limosilactobacillus reuteri* DSM 17938*, Limosilactobacillus fermentum* CECT 5716*, Bifidobacterium breve* M-16V*, Lactobacillus helveticus* R0052*, Bifidobacterium bifidum* R0071*, Bifidobacterium longum subsp. longum* BB536, *Bifidobacterium longum subsp. infantis* R0033, etc. (48–50). The conditions under which these probiotics are used in hypoallergenic formulas need to be researched in depth.

## 4 Physiological functions of probiotics on children's health

As for the concept of probiotics, the most widely used is the definition of Food and Agriculture Organization (FAO) and WHO (51): Probiotics are living microorganisms that, when ingested in sufficient quantities, produce one or more demonstrated physiological functional benefits to the host.

The intestinal flora of infants is mainly dominated by Bifidobacterium, which maintains a dynamic balance with the changes of environment, diet and lifestyle in the later period (52). When the balance of intestinal flora is broken, the disordered intestinal flora will affect the occurrence and development of many diseases (53). Probiotics can play a beneficial role by regulating the abundance of intestinal flora and its metabolites. Clinical studies have found that Probio-M8 can improve asthma symptoms by regulating intestinal flora (54). In addition to directly acting on intestinal flora, probiotics can also play a role by indirectly regulating metabolites of intestinal flora, such as short-chain fatty acids (SCFA) (55), bile acids, lipids, and neurotransmitters, so as to improve the health of the body.

Different probiotic strains and doses can have different effects on health outcomes, and no one-size-fits-all strain addresses all health outcomes. However, probiotic supplementation is thought to trigger numerous immunological benefits through signaling pathways, cytokine expression. Induction of cytokine secretion by probiotic bacteria exhibited strain specificity, and the response may also vary in the presence of different species of probiotic bacteria or a mixture of probiotic bacteria (56). Ingested probiotics have been reported to interact with enterocytes and dendritic cells, Th1, Th2, and regulatory T cells (Tregs) in the gut. It has been shown that probiotics can reduce inflammation by stimulating anti-inflammatory cytokines and reducing pro-inflammatory cytokines, which in turn modulate NK cell activity and inhibit Toll-like receptor (TLR), which in turn inhibits the nuclear factor-kappa B (NF-κB) pathway (57). Probiotics have been shown to suppress intestinal inflammation by down-regulating TLR expression. Depending on the type of TLR, reduced expression of TLR can lead to multiple benefits, such as reduced NF-κB activity and other proinflammatory expressions (58).

One of the important functional indicators to monitor the immunity enhancement of probiotics is its ability to inhibit the growth of pathogenic bacteria, which can secrete peptides and organic acids to inhibit the production of harmful substances such as amines and indole, thus having the effect of inhibiting pathogenic bacteria and relieving inflammation (59). Lactobacillus rhamnosus, the most common type of lactic acid bacteria in probiotics, can produce SCFA such as acetic acid, propionic acid and butyric acid through fermentation, thereby changing the osmotic pressure inside and outside the cells, forming an acidic environment, and having a synergistic effect on inhibiting pathogenic bacteria (60). Studies have also shown that probiotics can also secrete bacteriocins for wall membrane and intracellular to inhibit the growth of pathogenic bacteria (61–63). This is undoubtedly advantageous for children whose immune defense mechanisms are not well developed.

Due to the particularity of the children population and the complexity of CMA mechanism, in the industrial production of hypoallergic infant formula, how to improve the physiological function of probiotics is a key aspect of future research (64).

## 5 Mechanism of probiotics in the prevention and regulation of CMA in children

Since CMA children have shown differences in their gut microbiome composition (number and diversity of species), modulation of the intestinal microbiota seems a promising strategy for the control of allergic reactions. In addition, there are some evidences on the beneficial effects of probiotics on the natural history of CMA, recovery from CMA and the appearance of other allergic manifestations in pediatric age (65–68). Hence, there is increasing interest in the use of probiotics for the prevention and treatment of food allergies.

Based on the recent studies on the effects of probiotics and their metabolites on CMA in children, we speculate that probiotics may play an important role in CMA by promoting the early establishment of intestinal microecological balance, regulating the body's immunity, and enhancing the function of intestinal mucosal barrier. In addition, lactic acid bacteria, as one of the important probiotics, also have the potential to destroy allergen epitopes and thus reduce milk sensitization.

### 5.1 Promote the early establishment of intestinal microecological balance

The human microbiota is a complex microbial ecosystem composed of commensal, symbiotic, and pathogenic microorganisms that can be found in the gut, skin, oral cavity, nasal passages, and urogenital tract (69, 70). Early microbiota establishment is essential for proper immune development and is beneficial for overall health status (71). The colonization of the gut is a dynamic process that is thought to begin at the fetal stage, progressing through an ecologically ordered succession of species until reaching a steady and balanced composition (which occurs approximately 1,000 days after birth) (69, 70, 72–74).

Clinical studies of eHF supplemented with probiotics showed improved symptoms in infants with CMA (75–78). The addition of *Bifidobacterium* and LGG is beneficial to the early establishment of intestinal microecological balance in children with CMA (79). Candy et al. (80) showed that AAF including a prebiotic blend of fructo-oligosaccharides and the probiotic strain Bifidobacterium breve M-16V improves gut microbiota in non-IgE-mediated allergic infants. Canani et al. (81) found LGG-supplemented casein formula could cause enrichment of butyric acid-producing bacteria in gut for infants with CMA, thereby promoting tolerance and reducing the risk of allergy. Yanru (82) studied the structure of intestinal flora and SCFAs in feces of children with CMA and found that the presence of *Clostridium* and *Firmicutes* was related to infant's CMA, and the composition of SCFAs in feces of children was significantly different.

Probiotics can also promote the establishment of children's intestinal microecological balance by resisting pathogen colonization, because they may temporarily occupy the vacant functional niche in the resident microbiota and secrete reactive oxygen species to inhibit pathogen growth, thereby preventing opportunistic infections and reducing the occurrence of allergies (58).

### 5.2 Regulating the body's immunity and alleviating the allergic response

The addition of probiotics to infant formula to assist the management of CMA has become a research hotspot, this indicates that probiotics can regulate the body's immunity. As reviewed by Servin, different *Lactobacilli* and *Bifidobacteria* strains were reported to be capable of stimulating immune cells to secrete cytokines or shifting the Th2-type response back to a Th1-type response (83, 84). Song et al. (85) found that Lactobacillus rhamnosus2016SWU.05.0601 regulated immune balance in ovalbumin-sensitized mice by modulating expression of the immune-related transcription factors and gut microbiota, decreasing the levels of Th2 and Th17 but increasing the levels of Th1 and Treg cytokines.

Zhang's study indicates that oral administration of Bifidobacteria has the capacity to suppress the skewed Th2 response in allergic mice, increasing the number of Treg and IL-10-positive cells and improve the impaired intestinal epithelial barrier function (86), and Inoue et al. (87) showed that Bifidobacterium breve M-16V modulated the systemic Th1/Th2 balance, suppressed the IgE production and reducing IL-4 levels by the *in vitro* and *in vivo* experiments. Lactobacillus casei strain Shirota (LcS) was administered intraperitoneally to ovalbumin-specific T cell receptor transgenic (OVA-TCRTg) mice, which increased IL-12 levels, decreased IgE and IgG1 levels, and restored the Th1/Th2 balance (88). The same immunological responses were also induced by Lactobacillus plantarum L-137 which could stimulate IL-12 production and reduce serum IgE and IgG levels (89).

The immunomodulatory effect of LGG on CMA has been extensively studied. Incidence of allergic symptoms decreased in children with CMA after taking extensively hydrolyzed casein formula (EHCF) containing LGG not only in mice but also in humans (90, 91). It has been reported that supplementing EHCF with LGG is more effective compared to EHCF alone in reducing CMA (44, 92). Similar results were found in another study by Thang et al., who used 3-week-old newly weaned Balb/c mice with adjuvant-free LGG sensitization to simulate CMA (93). In LGG-treated mice, Th2 responses were suppressed, resulting in remarkably lower hypersensitivity scores and CMP-specific IgG1 levels, and Th1 responses were promoted, resulting in increased levels of IFN and CMP-specific IgG2a (94). Moreover, lactic acid bacteria and its surface molecules can also affect the production of immune cells and cytokines (95). Therefore, the use of lactic acid bacteria fermentation to reduce milk sensitization has a good prospect (96–98).

Probiotics can also regulate the inflammatory signaling of intestinal epithelial cells to alleviate allergy. NF-κB and mitogen-activated protein kinase (MAPK) are two important inflammatory pathways. Studies have found that probiotics can inhibit the activation of NF-κB. The lactic acid bacteria could inhibit the phosphorylation of p38 MAPK and p65 NF-κB to mediate inflammatory responses (99). L. acidophilus L-92 could activate Th1 and Treg cells by participating in MAPK and NOD-like receptor pathways (100). In addition, DeMuri et al. (101) found that Lactobacillus acidophilus NCFM/Bifidobacterium lactis Bi-07 may alter inflammation by decreasing expression of E-selectin. Li et al. (102) indicates that Bifidobacterium breve M-16V may alter the gut microbiota to alleviate the allergy symptoms by IL-33/ST2 signaling. Wang et al. (103) showed that surface layer protein (Slp) of Lactobacillus acidophilus NCFM prevents TNF-α-stimulated cell apoptosis, as well as inhibits IL-8 secretion via inhibiting NF-κB activity, thereby exerting its anti-inflammatory activity. Chen et al. (104) found that LGG could effectively alleviate the allergic response, restore the levels of HIS, IgE, MCP, MCT, specific IgG, specific IgG1, specific IgG2a, and other inflammatory factors, and restore CD4+ T cell infiltration and the status of intestinal villi.

### 5.3 Enhance intestinal mucosal barrier function

The mucus layer of the intestinal mucosa is a mechanical barrier against pathogens (105). The intestinal mucosal immune system is the most complex part of the body's immune system. Intestinal commensal bacteria can stimulate the development and maturation of the intestinal mucosal immune system in the early stage, activate Th1 immune response, and inhibit IgE production to prevent allergic reactions. Moreover, research have shown that probiotics can enhance the host intestinal immune barrier and improve the immune regulation ability of Treg cells in the immune system (106). Colonized lactic acid bacteria can enhance the tight junction between epithelial cells, reduce intestinal permeability, support intestinal barrier function, and thus reduce the stimulation of allergens (107, 108). LGG could produce both a biofilm that can mechanically protect the mucosa, and different soluble factors beneficial to the gut by enhancing intestinal crypt survival, diminishing apoptosis of the intestinal epithelium, and preserving cytoskeletal integrity. Because the polysaccharides and pili present on LGG surface allow it to adhere to and temporarily colonize the intestinal mucosa (109).

### 5.4 Destruction of allergen epitopes thereby reducing CMA

As one of the important probiotics, lactic acid bacteria can not only regulate the composition of intestinal flora to play an immunomodulatory function or produce a variety of stimulus signals to activate immune cells, thereby triggering systemic immune response (110). On the other hand, lactic acid bacteria have a complex protease system (111), which can produce peptidase and protease to hydrolyze milk protein, destroy allergen epitopes (112), and thus reduce milk allergy (113).

Therefore, probiotic use not only emerges as a safe microbiological strategy in pediatrics for the promotion of intestinal immunity, but also becomes an important research direction for future CMA management.

## 6 Overviews in the application of probiotics in hypoallergenic infant formula

The application of probiotics to modulate the gut microbiome-immune axis to alleviate CMA has become a research hotspot. However, the mechanism by which the gut microbiota regulates CMA and the efficacy of probiotics are still in the preliminary exploration stage, and there are no clear and specific conclusions (114). Therefore, it is very important to locate specific strains in hypoallergenic infant formula.

Because of the different target proteins of hydrolysis, hydrolyzed infant formula is divided into hydrolyzed casein formula and hydrolyzed whey formula. However, some brands of pHF or eHF still have allergen B cell epitopes and can cause allergic reactions (115). In recent years, the combination of hydrolyzed proteases and probiotics has developed hypoallergenic milk protein hydrolysates, which have a broader application field. Probiotics have been shown to be beneficial in reducing symptoms in allergic patients, and adding probiotics to hypoallergenic infant formula is an innovative way to prevent and treat CMA (116). If a certain amount of LGG is added to eHF, it will bring many benefits to the infant, LGG more quickly induces the tolerance of infants with CMA, reduces the incidence of allergic dermatitis in infants, improves inflammation in the intestine to a certain extent, but also improves the recovery of allergic colitis (117). However, the European Society of Pediatric Nutrition and the Society of Gastroenterology believe that these new infant formulas are still not entirely satisfactory because the real safety of the probiotics added to the formulas has not been fully evaluated (118).

The research on the anti-allergic mechanism of probiotics will be carried out through *in vitro* and *in vivo* experiments and establish a safety evaluation mechanism is the key research content in the future. Moreover, for the research and development of probiotic hypoallergenic formulations, the specific probiotics used alone or in combination, the timing of the start and end of treatment, and the appropriate dose are needed to be determined deeply.

## 7 Suggestions and prospects

For the management of CMA in children, probiotics combined with hypoallergenic infant formula to establish immune tolerance are recommended as the main route in the future. But the data on probiotics themselves as a CMA prevention strategy are imperfect. In-depth exploration of the mechanism of probiotics regulating CMA is the focus of current research.

The application of probiotics as functional ingredients in hypoallergenic infant formula by major brands in the dairy industry has become a research hotspot. However, the health effect of probiotics has high strain tolerance, and infants with different constitutions, different genetic backgrounds and different intestinal flora should be different, and the research on probiotics can be located on the individual strains. At present, researchers have evaluated the safety and the efficacy of EHCF with LGG by randomized, double-blind trial, and the results showed that EHCF + LGG could be tolerated by the vast majority of IgE-mediated CMA children, and that the step-down approach from AFF to EHCF + LGG could promote a faster acquisition of immune tolerance (119). Although the use of probiotics is beneficial to promote immune regulation and alleviate clinical symptoms, more methodologically based and homogenized research is needed to more specifically study each type, dose, and time of probiotic supplementation for the establishment of definitive care protocols.

An in-depth comparison of the mechanism of action of probiotics added to whey protein hydrolysate vs. casein hydrolysate to reduce CMA. Based on clinical data or the real-world evidence, the mechanism of probiotics in hypoallergenic formula powders was studied. Nevertheless, there is an increased risk of functional gastrointestinal disorders among infants with CMA which could be reduced among those fed with EHCF+LGG (120), and further research is required to fully elucidate the mechanism of action of the probiotics.

## Author contributions

ML: Conceptualization, Data curation, Formal analysis, Investigation, Methodology, Project administration, Visualization, Writing—original draft. CYJ: Conceptualization, Funding acquisition, Methodology, Resources, Supervision, Project administration, Visualization, Writing—review & editing.
